# CAREGIVER TYPE AND ITS ASSOCIATION WITH MORTALITY AMONG OLDER ADULTS WITH DISABILITY IN CHINA

**DOI:** 10.1093/geroni/igz038.299

**Published:** 2019-11-08

**Authors:** Chenkai Wu, Haoxue Li, Xiaoting liu, honglin chen

**Affiliations:** 1 Duke Kunshan Unviersity, Kunshan, Jiangsu, China; 2 Zhejiang University, Hangzhou, Zhejiang, China; 3 Fudan University, shanghai, China

## Abstract

A large proportion of older adults in China needs assistance with activities of daily living (ADL). The quality of care for this population has gained growing attention. However, little is known about the patterns of their caregiver type or its association with mortality. We examined the patterns of primary caregiver type among community-dwelling older adults in China and to examine the association between caregiver type and mortality. Data were from the 2005, 2008, and 2011 waves of the China Longitudinal Health and Longevity Study. We included 437 married and 3,971 widowed participants. The average age was 96.7 years (SD=6.9) and 73.7% were female. For married persons, 55.1%, 25.2%, 12.1%, 2.3%, and 3.9% of the primary caregiver was spouse, son/daughter-in-law, daughter/son-in-law, grandchildren, and housekeeper. For widowed persons, 60.2%, 21.1%, 9.9%, and 5.9% of the primary caregiver was son/daughter-in-law, daughter/son-in-law, grandchildren, and housekeeper, respectively. Multivariable-adjusted model showed that, among married older adults, son/daughter-in-law and daughter/son-in-law as the primary caregiver was associated with 41% (95% confidence interval [CI]=6%-87%) and 67% (95%CI=10%-154%) higher mortality than spouse as the primary caregiver, respectively. For widowed persons, daughter/son-in-law and grandchildren as the primary caregiver was associated with 12% (95%CI=3%-20%) and 14% (95%CI=2%-24%) lower mortality than son/daughter-in-law as the primary caregiver, respectively. Majority of disabled older adults in China relied on their spouse and children to care for them. Type of primary caregiver was associated with death in both married and widowed persons. More resources need to be allocated to disabled Chinese older adults with poor survival outcomes.

